# Long-term risk of mental health problems in women experiencing preterm birth: a longitudinal study of 29 mothers

**DOI:** 10.1186/1744-859X-12-33

**Published:** 2013-10-31

**Authors:** Aud R Misund, Per Nerdrum, Stein Bråten, Are Hugo Pripp, Trond H Diseth

**Affiliations:** 1 Faculty of Health Sciences, University College of Oslo and Akershus, HIOA, P.O. Box 4, St. Olavs plass, N-0130 Oslo, Norway; 2 Department of Sociology and Human Geography University of Oslo, P.O. Box 1096, Blindern, 0317 Oslo, Norway; 3 Unit of Biostatistics and Epidemiology, Oslo University Hospital, Ullevål sykehus, P.O. Box 4950, Nydalen NO-0424, Oslo, Norway; 4 Department of Clinical Neurosciences for Children, Women and Children's Division, Oslo University Hospital, Rikshospitalet, P.O. Box 4950, Nydalen NO-0424, Oslo, Norway; 5 Department of Medicine, Institute of Clinical Medicine, University of Oslo, Problemveien 7, 0313 Oslo, Norway

**Keywords:** Preterm birth, Psychological distress, Anxiety, Depression, PTSD

## Abstract

**Background:**

Several studies have reported significantly higher stress levels, both short and long terms, among mothers giving preterm birth compared with mothers giving birth at term. Stress, however, is a psychological phenomenon that may present as anxiety, depression and/or trauma reactions. In this study, the long-term mental health outcomes and the prevalence of anxiety, depression and trauma reactions in women experiencing preterm birth were explored. Interactional, main effect variables and predictors were identified.

**Methods:**

Twenty-nine mothers of 35 premature children born before the 33rd week of pregnancy were assessed within 2 weeks postpartum (T0), 2 weeks after hospitalization (T1), 6 months post-term (T2), and 18 months post-term (T3). The standardized psychometric methods Impact of Event Scale (IES), General Health Questionnaire (GHQ) and State Anxiety Inventory (STAI-X1) assessed the maternal mental health outcomes.

**Results:**

The maternal mental health problems except state anxiety decreased from T0 to T1, but remained high and stable at T3. The prevalence of posttraumatic stress reactions (PTSR) and posttraumatic stress disorder (PTSD) at T0 and T3 was 52% and 23%, respectively. We identified the time period between T0 and T1 to have a significant main effect on mental health outcomes. The predictors of higher levels of mental health problems were preeclampsia, previous psychological treatment, age, trait anxiety and infant's postnatal intraventricular haemorrhage. Bleeding in pregnancy predicted lower levels of mental health problems.

**Conclusions:**

The prevalence of maternal mental health problems remained high, emphasizing the importance of effective interventions.

## Introduction

Substantial psychological distress may be caused in mothers by the experience of giving preterm birth and successive experiences in the neonatal intensive care unit (NICU). Knowledge about long-term effects of a preterm birth is valuable to maternity units for implementation in the prevention of mental health problems postpartum. In early intervention programs involving examination of maternal psychological reactions to preterm birth on the maternity ward, knowledge of both short-term and long-term reactions is important in attaining the best treatment result for the mother, and for the relation to her newborn baby.

Several studies from the past decades have explored maternal mental health problems following preterm birth, both in the short and long terms [1-9]. Maternal postpartum stress reactions have been the main research focus in these studies [2,8-10], which have reported that women with preterm deliveries experience significantly higher levels of stress than women with term deliveries [2,8]. As a psychological phenomenon, stress may coexist with anxiety, depression and trauma reactions. The nature of the maternal psychological distress reported in many studies is therefore not clarified [4-6]. Although the co-morbidity of anxiety and depression in posttraumatic stress reactions (PTSR) is well known, the knowledge of PTSR following preterm childbirth is still limited [6]. Some of the studies have focused on depression [3,11-13], others on depression, distress, and anxiety [2,14-16]. Few studies, however, have explored maternal trauma reactions following preterm birth [4,6,7,17-22].

Why 10% of all women have severe traumatic stress responses to birth and only 1% to 2% develop posttraumatic stress disorder (PTSD) after giving birth is still not fully explored [23,24]. Subjective distress in labor and obstetrical emergencies, as well as infant complications, psychological difficulties in pregnancy and previous traumatic experiences have been identified as risk factors [25]. Both term and preterm deliveries may involve certain features such as intense fear, helplessness, pain and loss of control that might predispose for traumatic stress reactions [26,27]. One study reported a prevalence of 49% of significant trauma reactions 1 year following preterm delivery [7]. Other studies have found a correlation between traumatic stress reactions and disturbances in mother-child interactions. Further, the intensity of an unresolved trauma may have a mediating effect on later sleep and eating problems in the preterm infant [1,28]. The studies of mental health problems following preterm delivery, however, are in general small, non-randomized studies using different trauma instruments.

Parental mental health is found to be associated with children's cognitive, emotional, social and physical development [29]. Several studies of term babies have shown that maternal postpartum or chronic depressive symptoms correspond with poorer cognitive and behavioural child performance [30-34]. Postpartum depression has frequently been investigated, and the predictors that have achieved the greatest amount of consensus are previous psychiatric disorder and higher percentage of psychiatric disorder in the family, as well as poor social support, marital problems, and higher levels of stress during pregnancy [26]. Predictors of maternal psychological distress following term birth are less explored [35,36]. In one study, predictors of maternal distress 5 years following term birth were identified as initial psychological distress, being single and having a low educational level [36]. Predictors of maternal mental health problems following preterm birth have only been explored in the studies of DeMier et al. on PTSD at 6 months postpartum, where infant maturity and infant complications were detected as significant predictors of maternal PTSD [37,38]. To our knowledge, the present study is the first to detect predictors since those of DeMier et al.

The primary aim of the present study was to explore long-term mental health outcomes in mothers experiencing preterm birth before 33 weeks of pregnancy and to identify interactional, main effect variables and predictors.

## Methods

### Subjects

From June 2005 to July 2008 in two periods of measurement, the psychological responses of 29 consecutive mothers of a total of 35 premature children born before the 33rd week of pregnancy at a highly specialized NICU at the National Hospital (Oslo University Hospital), Norway were assessed as follows: first, within 2 weeks after preterm childbirth (T0), median 11 days (4–30 days); second, within 2 weeks post-discharge from hospital (T1), median time after birth 2.7 months (0.2–4.7 months); third, at the infant's 6-month post-term age (T2), median time after birth 8.5 months (7.6–10.4 months); and fourth, at the infant's 18-month post-term age (T3), median time after birth 20.6 months (19.2–23.4 months). Mothers of severely ill babies that the medical staff estimated to have poor chance of survival and mother who are non-native speakers were not included. Medical charts and a semi-structured interview were used to collect data about the birth and socio-demographic information.

The mothers in this study were a homogeneous group with high scores on socio-demographic variables such as education, income and housing standard. Most of them were giving first-time birth late in their twenties or early thirties (Table 1). All lived with the child's father, and none reported any relationship problems. The prevalences of pregnancy infection, planned Caesarean section, acute Caesarean section and breech birth in these pregnancies and deliveries were 42.9%, 8.6%, 45.7% and 8.6%, respectively.

**Table 1:** Socio-demographic, physical characteristics and mental health variables in mothers given preterm birth and their children

	**Number**
Mothers	29
Age; mean (SD)	33.7 (4.3)
Education > 12 years; *n* (%)	26 (89.7)
Single parent; *n* (%)	0 (0)
Unemployed; *n* (%)	4 (13.8)
Previous psychological treatment; *n* (%)^a^	8 (27.6)
Chronic illness; *n* (%)^b^	2 (6.9)
Total no. of children; mean (SD)	1.7 (0.8)
Previous pregnancies; mean (SD)	1.1 (1.5)
Previous childbirths; mean (SD)	0.5 (0.7)
First-time mothers; *n* (%)	18 (62.1)
IVF pregnancy; *n* (%)^c^	8 (27.6)
Bleeding in pregnancy; *n* (%)	19 (65.5)
Preeclampsia; *n* (%)	4 (14.3)
Caesarean; *n* (%)	17 (58.6)
Children, *n* (%)	35
Girl	17 (48.6)
Boy	18 (51.4)
Twin	14 (40)
Gestational age (weeks)	
Median (range)	29 (24–32)
Mean (SD)	28.5 (2.6)
Birth weight (kg)	
Median (range)	1.2 (0.6–2.0)
Mean (SD)	1.2 (0.4)
Apgar score	
At 1 min; median (SD)	6.3 (2.3)
At 5 min; median (SD)	7.6 (2.0)
At 10 min; median (SD)	8.3 (1.0)
Oxygen supply > 28 days; *n* (%)	19 (54)
IVH grade	
1 and 2; *n* (%)^d^	5 (14.3)
3 and 4; *n* (%)^d^	2 (5.7)

Assessment at 2 weeks postpartum (T0). ^a^Everyone who had been in psychotherapy as a child or as an adult was registered; ^b^diabetes and Crohn's disease; ^c^*in vitro* fertilization**/**assisted fertilization; ^d^intraventricular haemorrhage following birth.

The babies in this sample had relatively high Apgar mean scores. Only 23% of the babies needed mechanical ventilation for more than 24 h, and 6%, 11% and 17% of the babies had surgery, infections and patent ductus arteriosus after birth, respectively. The neonatal intensive care unit had applied several aspects of the newborn individualized developmental care and assessment program (NIDCAP) in their care of preterm babies and in their parental supervision and support [39]. Parents were offered psychological care during the hospital stay. Mothers with serious mental health reactions were referred for psychological treatment following discharge from the hospital.

### Assessments of maternal mental health problems

Maternal mental health problems were assessed using the standardized psychometric instruments Impact of Event Scale (IES), General Health Questionnaire (GHQ), and State/Trait Anxiety Inventory (STAI-X1/*X*2). The 15-item version of the Impact of Event Scale [40,41] was used to assess behavioural aspects of distress. Clinically important stress-related cognition and behaviour were defined as an IES score ≥19. In this study, the stress factor was defined as ‘preterm childbirth’. The IES-15 has two subscales measuring symptoms of intrusive psychological distress (seven items) and avoidant cognition and behaviour (eight items). The scoring range for each item is 0 (not at all) to 5 (very much). A subscale score of 0–8 usually denotes minor responses, 9–19 moderate responses and scores ≥20 denote severe responses. IES has been thoroughly validated and is one of the key psychometric assessment methods in traumatic stress research [40,41].

The General Health Questionnaire [42] is a widely used screening instrument for assessing the presence of distress, psychopathology and overall well-being, showing acceptable and well-established reliability and validity. The GHQ-30 contains 30 items, and each question is answered on a four-point scale. The answers to each item may be treated both as Likert sum scores with weights (0-1-2-3) and a possible scale of 0–90, and as case sum scores with weights (0-0-1-1) and possible range 0–30 [42,43].

The Spielberger State Trait Anxiety Inventory (STAI-X1 and STAI-*X*2) [44] was used to assess maternal anxiety. STAI-X1 is a measure of state anxiety levels reflecting subjective feelings of tension, apprehension, nervousness and worry. STAI-X1 has a 20-item and a 12-item version, and both were used in our study. Both versions consist of items rated on a four-step scale (1-2-3-4) with a possible score range of 20–80 for the 20-item version and 12–48 for the 12-item version. Higher scores indicate more anxiety. Ten items from the 20-item version overlap in the two versions (item nos.: 1, 2, 3, 5, 7, 11, 12, 13, 14 and 15). A common ten-item STAI version was constructed for our analyses. For the ten-item version, clinically important state anxiety was defined as a STAI score ≥20 (corresponding to ≥ 40 for the 20-item version). STAI-*X*2 is a measure of trait anxiety that refers to individual differences in anxiety proneness, i.e. in the tendency to see the world as dangerous and threatening, and the frequency with which anxiety states are experienced. It consists of 20 items, and the scoring range is 20–60. Clinically important significant trait anxiety was defined as ≥ 40. The STAI-X1 and *X*2 are reliable and widely used self-evaluation questionnaires employed in several studies with similar populations [44].

To explore the prevalence of anxiety, depression and PTSR/PTSD in particular, a tentative clinical diagnosis based on the clinical diagnostic guidelines in the ICD-10 Classification of Mental and Behavioural Disorders [45] was assessed by a psychiatrist (last author). The assessment was based on all information available in a clinical perusal of the psychometric self-reports IES, GHQ and STAI of each of the 29 preterm mothers, and blinded to the physical and socio-demographic characteristics of the mothers and their children.

### Statistical methods

Values of continuous variables are presented as means (SD) or if skewed, as median and range. Categorical variables are given as proportions and percentages. A random intercept linear mixed model for repeated measurements with fixed effects of follow-up time was used. Correlations between continuous variables were assessed with Pearson or Spearman correlation coefficients as appropriate. Linear regression analysis was used to identify possible predictors of mental health and psychological distress at the last follow-up time point using forward stepwise variables selection with *p* value < 0.05 as inclusion criteria. A careful check of the model assumptions, including an investigation of residual plots, was conducted. All analyses were performed in SPSS version 19. Two-sided statistical tests were applied, and a 5% statistical significance level was chosen.

### Ethical consideration

Written informed consent was obtained from participants prior to the study start. The study protocol was approved in May 5, 2005 and April 19, 2007 by the Norwegian National Committee for Research Ethics (S-05068 and S-07096b) and April 1, 2005 and March 12, 2007 by the Data Inspectorate (12360 and 07/1088). The study protocol was carried out in accordance with the Declaration of Helsinki.

For ethical reasons, the mothers who reported clinically significant mental health problems were assessed for and referred to adequate psychological treatment. In this study, we detected clinically important mental health problems in mothers at four points, from the time of birth to two years postpartum. A no referral procedure could have caused unnecessary suffering for both mother and child.

## Results

At T0, 29 of the 34 mothers (85.3%) who met the inclusion criteria were included in the study (Table 1). Five mothers refused to participate in the study. Their reasons were lack of energy or mental capacity, the burden of a premature baby at NICU, lack of time for being interviewed and refusal to be videotaped. None of the mothers who refused to participate differed from the participants regarding socio-demographic background or characteristics of the child's medical condition. Two of the mothers at T1 and one of the mothers at T4 withdrew from the study.

Fourteen of the mothers received psychological counselling at the hospital during their infants' NICU stay. Eleven of these mothers were referred for further psychological treatment after discharge from the hospital. Altogether, 20 mothers who met the criteria of clinical diagnosis were offered referral to adequate psychological treatments such as psychotherapy, psychopharmaceutical treatment and mother-infant interactional treatment following discharge from the hospital. Five of the mothers refused referral for psychological treatment. Fifteen of the mothers accepted referral and received adequate psychological treatment following discharge from the hospital.

### Long-term maternal mental health problems

**Figure 1:**
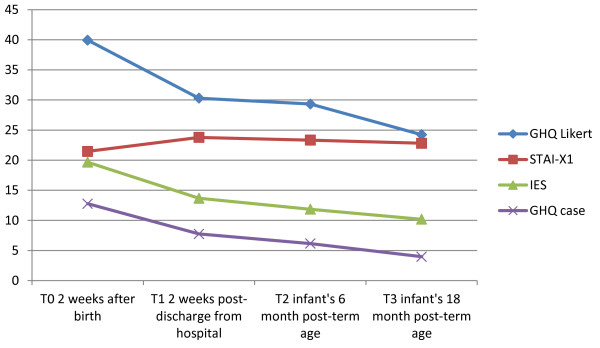
**Development of mean psychological distress, anxiety and trauma-related stress reactions.***N* **=** 29 mothers who have given preterm birth.

The IES mean sum score, the GHQ Likert mean sum score and the GHQ case mean sum score all showed significant reduction from T0 to T1, except for the STAI-X1 mean sum scores that showed significant increase. From T1, we found a small non-significant reduction of all four instruments to T2 and T3 (Table 2, Figure 1).

**Table 2:** Long-term trauma-related stress reaction, psychological distress, and anxiety in mothers following preterm birth

	***N***	**Means (SD)**	**Estimated marginal means**	**Standard error**	***p *** **value by time**
IES total (0–75)					
T_0_	29	19.7 (10.8)	19.7 (15.5, 23.8) a	2.1	*p* < 0.001
T_1_	27	13.7 (11.5)	13.0 (8.8, 17.3) b	2.1	
T_2_	27	11.9 (9.7)	11.2 (7.0, 15.5) b	2.1	
T_3_	26	10.2 (11.9)	10.1 (5.8, 14.4) b	2.1	
GHQ Likert (0–90)					
T_0_	29	40.0 (15.4)	39.9 (34.4, 45.5) a	2.8	*p* < 0.001
T_1_	27	30.3 (13.4)	29.9 (24.2, 35.6) b	2.9	
T_2_	27	29.3 (17.1)	28.9 (23.2, 34.7) b	2.9	
T_3_	26	24.2 (13.9)	23.7 (17.9, 29.5) b	2.9	
GHQ case (0–30)					
T_0_	29	12.8 (7.5)	12.8 (10.1, 15.4) a	1.3	*p* < 0.001
T_1_	27	7.7 (6.6)	7.5 (4.8, 10.2) b	1.4	
T_2_	27	6.2 (7.7)	5.9 (3.2, 8.6) b	1.4	
T_3_	26	4.0 (6.4)	3.7 (0.9, 6.4) b	1.4	
STAI-X1 (0–40)^a^					
T_0_	29	21.5 (2.8)	21.5 (20.5, 22.4) a	0.5	*p* = 0.001
T_1_	27	23.8 (2.7)	23.8 (22.8, 24.7) b	0.5	
T_2_	27	23.3 (2.3)	23.3 (22.4, 24.3) b	0.5	
T_3_	26	22.8 (1.8)	22.9 (21.9, 23.8) ab	0.5	

Means (SD) and estimated marginal means (95% confidence intervals) with standard errors from linear mixed model statistical analysis of the IES, GHQ and STAI-X1 at 2 weeks after birth (T0), 2 weeks post-discharge from hospital (T1), infant's 6-month post-term age (T3) and infant's 18-month post-term age (T4). Same letter of estimated marginal means within each instrument at different observation times indicates that the estimates are not statistically different at *p* < 0.05 from each other. For example, for three given estimates with a, ab and b, the first a is only significantly different from the third b, and the middle one ab is not significantly different from the others (a or b). ^a^Ten-question version of STAI-X1.

We detected a statistically significant effect of time between T0 and T1 on the IES sum score (*p* < 0.001), GHQ Likert and GHQ case sum scores (*p* < 0.001), and the STAI-X1 sum scores (*p* = 0.001) (Table 2).

**Figure 2:**
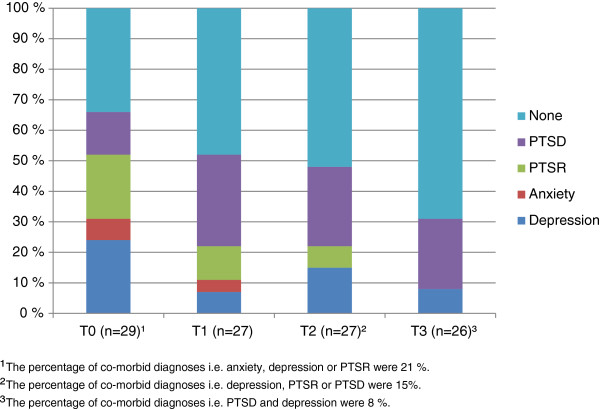
**The appearance of tentative depression, anxiety, PTSR and PTSD diagnoses among the mothers.** Only the first diagnosis was recorded if there were co-morbidities.

The tentative diagnoses based on all information available in a clinical perusal of the psychometric self-reports revealed that 66%, 52%, 48% and 31% of the mothers fulfilled the criteria for a psychiatric diagnosis at T0, T1, T2 and T3, consecutively. However, the prevalence of PTSR (first or only diagnosis) was reduced from 21% at T0 to 0% at T3. PTSD on the other hand increased from T0 (14%) to T1 (30%), and to T3 (23%). The prevalence of PTSR and PTSD, when co-morbid diagnoses were included, was 52%, 41%, 33% and 23% at T0, T1, T2 and T3, respectively. Depression decreased from 24% at T0 to 8% at T3 (Figure 2).

### Associations between physical variables and maternal mental health problems

Higher maternal mental health problems were significantly associated with the mother's age, previous psychological treatment, parity, trait anxiety, preeclampsia, acute Caesarean section and infant's IVH grade 1 or 2. Other physical variables such as vaginal delivery, planned Caesarean section, bleeding in pregnancy and the newborn infant on CPAP for less than 5 days were significantly associated with lower maternal mental health problems (Table 3).

**Table 3:** Significant correlations between socio-demographic, physical, and means of maternal mental health outcome variables

	**IES**	**GHQ Likert**	**GHQ case**	**STAI-X1**
Mother				
Age	43*			
Previous psychological treatment	0.37*	0.53**	0.36*	
Parity	0.36*	0.49**	0.38*	
Trait anxiety (STAI-*X*2)	0.35*			0.56**
Pregnancy/birth				
Preeclampsia	0.46**	0.56**	0.48**	
Vaginal delivery		−0.44**	−0.44**	
Caesarean		0.41*	0.41*	
Acute Caesarean			0.35*	
Planned Caesarean				−0.45**
IVF	0.34*			
Bleeding in pregnancy		−0.57**	−0.52**	
Child				
CPAP less than 5 days			-.36*	
IVH^a^	.37*			

Assessment at the infant's 6-month post-term age (T2) and the infant's 18-month post-term age (T3). Pearson's correlation coefficients (*p* < 0.05) are reported. *N* = 33. *Correlation is significant at the 0.05 level (two-tailed); **correlation is significant at the 0.01 level (two-tailed). ^a^Intraventricular haemorrhage, grade 1 or 2 shortly after birth.

### Predictors of maternal mental health problems

In the multiple linear regression analyses with forward stepwise variable selection (Table 4), we revealed three predictors for the IES: ‘preeclampsia’, ‘IVH grade 1 or 2’ and ‘mother's age’ explaining 41% of the variance in the IES. ‘Bleeding in pregnancy’ and ‘previous psychological treatment’ were the two significant predictors of GHQ Likert sum scores and explained 42% of the variance. The coefficient of bleeding in pregnancy was negative (*B* = −7.57), i.e. mothers with bleeding in pregnancy had 7.57 lower mean GHQ Likert sum scores compared to those without bleeding. The ‘maternal trait anxiety’ measured by STAI-*X*2 was the only statistically significant predictor for STAI-X1 and explained 29% of the variance in the STAI-X1.

**Table 4:** Multiple linear regression analyses predicting maternal mental health outcomes following preterm birth

**Dependent variable**	**Independent variable**	***B***	**95% CI**	**Beta**	***p *** **value**	***R***^**2**^_**adj**_
IES	Preeclampsia	5.75	(1.22, 10.27)	0.37	0.015	
	IVH^a^	11.34	(3.17, 19.50)	0.39	0.008	
	Mother's age	0.91	(0.18, 1.64)	0.36	0.016	0.41
GHQ Likert	Bleeding in pregnancy	−7.57	(−12.55, −2.59)	−0.44	0.004	
	Previous psychological treatment	13.79	(3.45, 24.13)	0.39	0.011	0.42
STAI-X1^b^	Maternal trait anxiety	0.23	(0.10, 0.36)	0.56	0.001	0.29

The mean of mental health outcomes assessed by IES, GHQ and STAI-X1 at the infant's 6- and 18-month post-term ages was used as outcome. Unstandardized (*B*) and standardized (beta) regression coefficients from multiple linear regression analyses after forward stepwise variable selection are reported. *N* = 33. ^a^The child was struck by intraventricular haemorrhage grade 1 or 2 shortly after birth; ^b^Ten-question version of STAI-X1.

## Discussion

In this study, we examined long-term maternal mental health outcomes following preterm birth. It is noteworthy that we revealed high and prolonged levels of maternal psychological distress, anxiety and trauma-related stress reactions. The levels remained high and stable after an initially significant decrease. In addition, our study revealed a main effect of time between T0 and T1 on maternal mental health outcomes. The prevalence of PTSR decreased from T0 to T3; PTSD showed a considerable increase from T0 to T1 and then stabilized toward T3. Altogether, 23% of the mothers reported PTSD, though only 8% of the mothers reported depression at T3. Preeclampsia, IVH, mother's age, bleeding in pregnancy, previous psychological treatment and maternal trait anxiety were detected as predictors for mental health outcomes in this study.

One striking result in our study is the significant effect of time between T0 and T1 on maternal psychological distress, anxiety and trauma-related stress reactions. Our findings correspond to some extent with the study of Holditch-Davis et al. on the effect of time on depression [5] but are contradictory with regard to state anxiety. It is interesting that their study revealed significant decrease in posttraumatic stress scores in the ‘high depressive symptom’ and ‘extreme distress’ classes in their study, which corresponds with our results of significant decreased IES scores from T0 to T1.

We found higher levels of maternal psychological distress, anxiety and trauma-related stress reactions following preterm birth, both short and long term, compared to studies of mothers giving birth at term and mothers giving birth to children with malformations. Our results correspond with the results of another study [46]: the study of Kersting et al. also reported a marked difference between preterm and term outcomes in IES and STAI-X1 [6]. However, our results in IES are lower than their results at 6 months (T2) and 18 months (T3). Another study reported mean sum scores in IES, GHQ Likert and STAI-X1 for mothers giving birth at term that were ordinarily 48%, 38% and 34% lower, respectively, at points of measurement comparative to T0, T1 and T2 in our study [36]. One study of psychological distress in parents of children born with malformation reported 19% lower scores in IES at T0, but equivalent results at T1 and T2 [47,48]. Our findings are interesting and raise questions about effective treatment programs for maternal mental health problems following preterm birth. Altogether, 18 of 29 mothers included in our study received psychological counselling in the hospital or psychological treatment following discharge from the hospital. Further research on effective treatment programs for this group is necessary.

Interestingly, our study showed a decrease in PTSR, anxiety and depression, but an increase in PTSD from T0 to T3. We revealed 23% higher prevalence of PTSD at T3 in our study, and our results correspond with the findings in the study of Engelhard et al. [4]. Our PTSR/PTSD results also correspond with the Jotzo and Schmitz study, but the latter reported higher levels of traumatic symptoms (77% at T0, and 49% at T2). However, our results were higher than theirs at T3 (12.5%) [20].

The prevalence of anxiety and depression that we found corresponds with the results of other studies [12,13,15] and contrasts with those of studies that have reported higher prevalence of depression at T0 and T1 [3], and a decrease to zero at T2 [15].

We found that higher maternal mental health outcomes were associated with ‘acute Caesarean section’, and lower maternal health outcomes were correlated with ‘planned Caesarean section’ and ‘vaginal delivery’. Our results seem to be inconsistent. However, both planned Caesarean section and vaginal delivery may present more control and less distress in labor for the mother, while an acute Caesarean section may be experienced as more distressing with less time to prepare for the critical preterm birth event.

Another striking result in our study was the physical predictors of maternal mental health outcomes. ‘Preeclampsia’ and ‘bleeding in pregnancy’ are perinatal complications that we found to predict posttraumatic stress responses in IES and general psychological distress in GHQ, respectively. One should note that bleeding in pregnancy reduced the GHQ outcome. There is no obvious explanation for this result in our study. Is it possible, however, to provide an existential explanation for why bleeding in pregnancies contribute to lower psychological distress in GHQ? Could it be that bleeding in pregnancy is experienced by the mother as a near-loss event early in pregnancy, leaving her with the frightening impression that her child is at risk? Given such an enduring impression of threat, the mother can be expected to be prepared for incidents such as a preterm birth. It is possible that the preparation of the mother for possible risks explains the low psychological stress outcome. Our detection of preeclampsia as a predictor of posttraumatic stress might correspond with the findings of Blom et al. that certain perinatal complications like preeclampsia, hospitalization, emergency caesarean and foetal distress predicted higher depression outcomes in a normal population sample [11]. Depression is often a co-morbidity of psychological trauma, but posttraumatic stress symptoms were not examined in their study.

In addition, we detected that ‘intraventricular haemorrhage (IVH) grades 1 and 2’ was a predictor of maternal posttraumatic stress responses in IES. Only two children in our sample were struck by IVH grade 3 or 4 following birth. The analysis of the impact of the most severe IVH was limited by our small sample size. Our result, however, corresponds to some extent with those of Singer et al. who found that maternal distress following preterm birth of a very low-birth weight child depended on the medical risk status, age and developmental outcome of the child [2]. Our finding also corresponds with the studies by DeMier et al. that postnatal complications in infants predicted maternal PTSD symptoms [37,38].

Other predictors of posttraumatic stress responses in IES, general psychological distress in GHQ, and state anxiety in STAI in our study were ‘mother's high age’, ‘previous psychological treatment’ and ‘trait anxiety’, respectively. Our results correspond with one of the predictors of postpartum depression, previous psychiatric disorder [26], and with the results from postpartum research showing trait anxiety predicts posttraumatic stress and anxiety [49].

### Strength and limitations

The strength of the present study is the longitudinal design. The participants came from well-defined geographic areas and were included consecutively in the study, thus minimizing selection bias. However, the exclusion of severely ill babies with very small chance of survival could affect selection bias. On the other hand, the impact of maternal grieving following the death of a preterm baby would represent a significant difference from our study group, though grieving mothers would have been interesting as a comparison group. The response rate is high both for the case and the psychometric instruments (IES, GHQ and STAI-X1) used in our study. These instruments are commonly used and have been validated in the research literature. We have assessed several important aspects of mental health problems, like psychological distress, anxiety and trauma-related stress, and also assessed tentative clinical diagnosis for the prevalence of mental health problems.

The present study describes a small preterm group of mothers with higher educational attainment, greater age, higher rate of IVF and higher socioeconomic status than would be found in a typical population of mothers who deliver preterm in our country. In our sample, 27.6% had been through IVF treatment to become pregnant. The moderating role of this treatment on the mother's psychometric outcome has not been examined. The homogeneity of the group in terms of socio-demographic background and distress related to it represents a limitation in this study. On the other hand, our results were controlled for high-risk socio-economic background variables as no one in our study group reported any socio-economic problems in the semi-structured interview we used for data collection.

Ethical considerations called for a referral procedure to meet the need for treatment when severe maternal mental health problems in the preterm mothers were detected. It is a limitation that we cannot assess to what extent the psychological treatment influenced our results.

## Conclusions

In this study, we examined the long-term maternal mental health outcomes in mothers experiencing preterm birth before the 33rd week of pregnancy. After a significant decrease from T0 to T1, we found high and prolonged levels of maternal mental health problems following preterm birth. One interesting result in this study is the high prevalence of PTSD of 28 % at the last point of measurement, T3. This result is particularly interesting since the participants in need of psychological treatment were consecutively referred for adequate psychological treatment throughout the study period. Even though we found a decrease in PTSD from 30% at T1 to 23% at T3, the decrease is not convincing regarding the expected effect of treatment. We revealed a prevalence of 28% PTSD at T3 which indicates that the most severe traumatic reactions remain active long term and consequently need long-term follow-up. We conclude that research in this field should be given priority as we need further knowledge on parental mental health problems following preterm birth and on how parental mental health problems influence early preterm parent-infant interactions. In addition, precautions in prenatal, natal and neonatal care, and effective treatment programs at the NICU are essential for preventing difficulties in parent-infant relations.

## Competing interests

The authors declare that they have no competing interests.

## Authors’ contributions

The ARM initiated the study and collected and organized the data. ARM and AHP performed the statistical analyses. ARM, PN, SB, AHP and THD wrote the article. All authors read and approved the final manuscript.
